# RMapAlign3N: fast mapping of 3N-Reads

**DOI:** 10.1093/bioadv/vbaf164

**Published:** 2025-07-09

**Authors:** Andre Müller, Alexander Wichmann, Felix Kallenborn, Andreas Hildebrandt, Bertil Schmidt

**Affiliations:** Institute of Computer Science, Johannes Gutenberg University, Mainz 55128, Germany; Institute of Computer Science, Johannes Gutenberg University, Mainz 55128, Germany; Institute of Computer Science, Johannes Gutenberg University, Mainz 55128, Germany; Institute of Computer Science, Johannes Gutenberg University, Mainz 55128, Germany; Institute of Computer Science, Johannes Gutenberg University, Mainz 55128, Germany

## Abstract

**Summary:**

Nucleotide conversion sequencing techniques are frequently used for the detection of various types of chemical modifications at nucleotide level. However, mapping of chemically treated reads to large reference sequences that contain only three nucleotides can be highly compute-intensive. We present RMapAlign3N—an efficient yet accurate tool for mapping of 3 N-reads to reference genomes or transcriptomes that leverages the power of modern multi-core CPUs. Our performance evaluation using real and simulated data shows that RMapAlign3N is faster and more scalable than prior CPU-based approaches including HISAT-3N, BSMAP, Bismark, and SLAM-DUNK for BS-seq and SLAM-seq data at competitive accuracy.

**Availability and Implementation:**

RMapAlign3N is open source software written in C++ and can be downloaded at http://github.com/muellan/rmapalign3n.

## 1 Introduction

Nucleotide conversion (NC) sequencing techniques are crucial for the detection of DNA or RNA modifications. They require corresponding molecules to undergo chemical treatment that change nucleobases, which in turn affects how nucleotides are read by next generation sequencing (NGS) platforms such as Illumina. We focus on 3 N conversions in which one of the four base letters (A, C, G, T) is replaced by a different one in the final sequence data if a specific type of modification is present or absent in the original biomelecule.

Prominent examples include BS-Seq (Bisulfite sequencing) and SLAM-seq (thiol (SH)-linked alkylation for metabolic sequencing of RNA). BS-seq features C-to-T conversions in the sequencing reads for unmethylated cytosine while leaving methylated or hydroxymethylated cytosine (5mC or 5hmC) unaffected. SLAM-seq introduces T-to-C conversions in the reads when the reverse transcriptase encounters an alkylated s^4^U residue during RNA to cDNA conversion.

Standard alignment algorithms such as Bowtie2 (Langmead and Salzberg 2012) are often not able to accurately align the generated NC reads to an unmodified reference since converted nucleotides would be treated as mismatches leading in turn to large fractions of incorrect or unmapped reads. As a consequence, several mapping tools have been developed specifically designed for NC reads. The three-letter strategy is a popular approach for mapping 3 N sequencing data. It is based on replacing the converted letter in both the reference sequence and the sequencing reads by the newly introduced letter (e.g. for BS-Seq all C’s are replaced by T’s). Afterwards, known aligners such as Bowtie2 or HISAT2 (Kim et al. 2019) are executed. Examples of aligners for BS-seq reads include Bismark (Krueger and Andrews 2011), BSMAP (Xi and Li 2009), and Arioc (Wilton et al. 2018), while SLAM-DUNK (Neumann et al. 2019) is designed for SLAM-seq data. Moreover, HISAT-3N (Zhang et al. 2021) is an extension of HISAT2 that can handle various types of NC 3 N reads including both BS-seq and SLAM-seq.

The methodologies employed by these tools are mostly based on first identifying candidate conversions with the help of *k*-mer based seeding or hashing techniques followed by alignment and analysis steps. Bismark aligns 3 N-converted reads to a 3 N-converted reference using Bowtie2 as underlying aligner; HITSAT-3N first builds an index of 3 N-converted references followed by a 3 N-read mapping stage that uses a graph FM index; BSMAP uses a hashing-based approach and bitwise masking; SLAM-Dunk relies on an interval tree data structure and NextGenMap (Sedlazeck et al. 2013) for alignment; Arioc performs a non-gapped spaced-seed alignment followed by a GPU-accelerated alignment step.

Although several aligners have been developed, they can still be highly time-consuming for large-scale datasets. As NC sequencing technologies further advance, finding more efficient solutions is of high importance to research.

In this paper, we present a new software tool for aligning 3 N reads called *RMapAlign3N* (Read Map Align for 3 N conversion techniques) based on combining a locality sensitive hashing (LSH) approach for efficient candidate identification with multiple sequence alignment (MSA) construction to effectively remove false positives.

Our performance evaluation shows that it can outperform HiSAT3N, BSMAP, and Bismark for mapping BS-seq reads by a factor of 4.9, 11.3, and 95.5, when executed on the same multi-core CPU at a similar alignment quality. Furthermore, RMapAlign3N outperforms SLAM-DUNK and HI-SAT3N for mapping SLAM-seq reads by a factor of 2.1 and 3.7. In addition, it scales better than other tested tools with respect to the number of utilized CPU cores.

## 2 Results

We compared the performance of RMapAlign3N to HISAT-3N (v.2.2.1), BSMAP (v.2.90), Bismark (v.0.24.2) for BS-seq reads, and HISAT-3N and SLAM-DUNK (v.0.4.3) for SLAM-seq reads on CPUs. Furthermore, we benchmarked Arioc (v.1.52) on a GPU. All CPU benchmarks were performed on a workstation with an AMD EPYC 7713P 64-core CPU and 512 GB of RAM using 128 threads. The GPU benchmarks were run on a workstation with two Intel Xeon Gold 6238 CPUs, 192 GB RAM, and an NVIDIA V100 GPU card.

We used the following data sets to evaluate alignment performance:

1. **BS-seq-sim:** 100 million 100-bp paired-end BS-seq reads with a 50% C-to-T conversion rate and a 0.2% per-base sequencing error rate simulated using Sherman (https://github.com/FelixKrueger/Sherman)
2. **BS-seq-real:** 78 million 125-bp real paired-end whole-genome BS-seq reads (SRA accession: SRR3469520)
3. **SLAM-seq-sim:** 100 million 100-bp single-end reads generated from 3’ regions of transcripts with a 2% T-to-C conversion rate and a 0.2% per-base sequencing error rate simulated using SLAM-DUNK
4. **SLAM-seq-real:** 45 million 100-bp real-world single-end SLAM-seq reads (SRA acccesion: SRR5806774)

The human genome GRCh38 was used as reference in all BS-seq tests and a human transcriptome from GENCODE GRCh38.p13 as reference in all SLAM-seq tests. All reads stemming from real experiments were trimmed with Trim Galore! to remove adaptors and all reads with a length less than 40-bp were discarded.

In addition to measuring runtimes and memory consumption for each tool, we determined the (unique) alignment accuracy (only for simulated datasets) and the (unique) alignment rate (for all datasets) as follows.


$$\begin{array}{c} (\text{unique})\;\text{align}\;\text{rate}=\frac{\#\;(\text{unique})\;\text{aligned}\;\text{reads}}{\#\;\text{reads}\;(\text{pairs})}\\ (\text{unique})\;\text{align}\;\text{accuracy}=\frac{\#\;(\text{unique})\;\text{correctly}\;\text{aligned}\;\text{reads}}{\#\;\text{reads}\;(\text{pairs})} \end{array}$$

Note that reads that are aligned to multiple locations are classified as correctly aligned if any of these candidate locations is correct. In case of unique alignment rate/accuracy only alignments with a single reported mapping location are considered. In case of multiple candidate locations with the same mapping score we select the first from the candidate list whose ordering is determined by the execution order and thus not deterministic.

**Figure 1.**
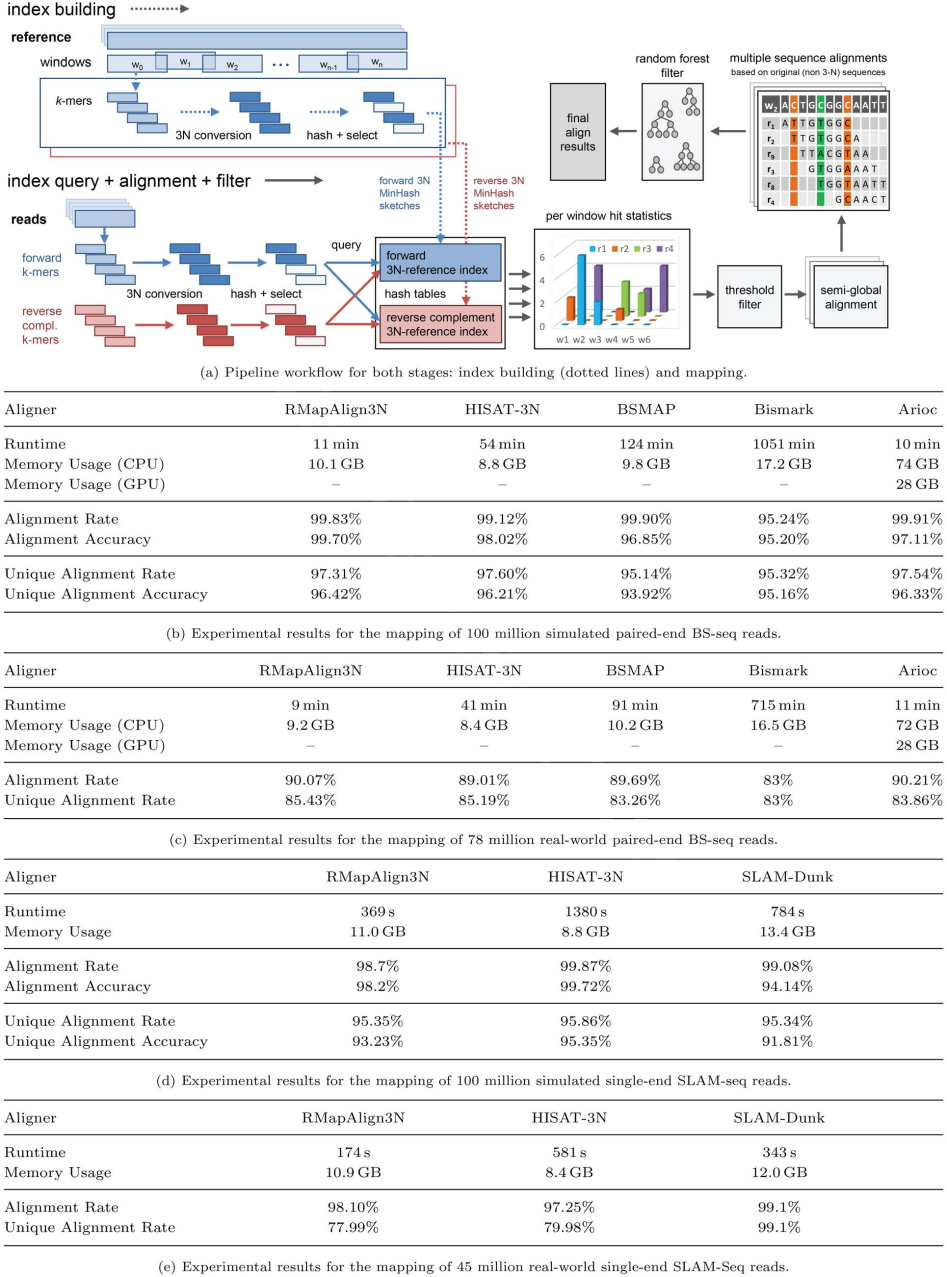
Workflow diagram and experimental results. (a) Pipeline workflow for both stages: index building (dotted lines) and mapping. (b) Experimental results for the mapping of 100 million simulated paired-end BS-seq reads. (c) Experimental results for the mapping of 78 million real-world paired-end BS-seq reads. (d) Experimental results for the mapping of 100 million simulated single-end SLAM-seq reads.

Figure 1b and c show the runtimes, memory usage, and (unique) alignment rate/accuracy for each tested tool for the simulated and real BS-seq data, respectively. RMapAlign3N is the fastest CPU-based tools in both tests with speedups of 4.9 (4.7), 11.3 (10.1), and 95.5 (79.4) using simulated (real) data over HISAT-3N, BSMAP, and Bismark, respectively. Furthermore, it exhibits the highest alignment accuracy for simulated data and the highest unique alignment rate for both real and simulated data. In addition, it achieves comparable runtime to GPU-accelerated Arioc for simulated data and even slightly faster runtime (while consuming almost an order-of-magnitude less memory) for real-world data being executed only on a CPU eliminating the need of using specialized hardware platforms.

Figure 1d and e show the runtimes, memory usage, and (unique) alignment rate/accuracy for each tested tool for the simulated and real SLAM-seq data, respectively. RMapAlign3N is the fastest tool in both tests with speedups of 3.7 (3.3), and 2.1 (2.0) using simulated (real) data over HISAT-3N, and SLAM-DUNK, respectively. SLAM-DUNK has the highest alignment rates but exhibits the lowest (unique) alignment accuracy of all tested tools. HISAT-3N shows the best alignment accuracy for SLAM-seq data with RMapAlign3N being second. The better performance of HISAT-3N could be explained by its use of a graph-based spliced alignment strategy.

In addition, we compared the thread scalability of different tools in Supplement Table 1 with the number of threads varying from 4 to 128. Overall, RMapAlign3N exhibits the best thread scaling behaviour with an efficiency of 42% for 128 threads outperforming HISAT-3n (32%), BSMAP (22%), and Bismark (15%).

## 3 Methods and implementation

RMapAlign3N is implemented in C++ using multi-threading targeting Linux workstations. It combines *MinHashing* (Broder 1997) of *k*-mers for fast candidate selection and inspection of MSAs in order to achieve both high speed and accuracy for NC read mapping. Our pipeline consists of the following steps and is illustrated in Fig. 1a.

1. 3N reference index building
2. Index querying
3. 3N-alignment and MSA construction
4. MSA evaluation
5. Result output

### 3.1 3N reference index building

We build two index databases from the reference sequence in a pre-processing step: one for the forward sequence and one for its reverse complement. Both indices are built with the desired nucleotide conversion applied, e.g. in the case of BS-seq, cytosine will be changed to thymine during index building. The indices are constructed using *MinHashing* to store information for each narrow region of a sequence in a compact way. This allows for efficient approximate match queries based on an approximation of the Jaccard index of feature sets derived from sequence segments.

First, the reference sequence is divided into windows of length *w* (by default *w *= 64) that overlap by k−1 base pairs. For each of these windows, all substrings of length *k* are generated (by default *k *= 16) and a hash function $h_{1}$ is applied to these *k*-mers. The resulting set of hash values, also called *features*, is sorted and the *s* (by default *s *= 16) lexicographically smallest values are selected. This reduced set of features is then stored in a hash table which maps features to reference sequence locations identified by both a sequence ID and a window ID. In order to achieve a good hash table bucket distribution that minimizes performance-degrading collisions, features are hashed again with a hash function $h_{2}$ before being inserted into the table. This subsampling scheme for fast non-exact sequence matching is largely based on our prior work for metagenomics (Müller et al. 2017). Similar hash-based sequence indexing and matching techniques are also discussed in (Alser et al., 2021, Kille et al. 2023).

### 3.2 Index querying

For each input read, we generate two 3 N reads, one forward and one reverse complement read, both with the selected 3 N-conversion applied. Converted reads are then queried against both 3 N reference indices thus producing four results per read. Duplicate mapping candidates are removed in the following MSA stage.

Querying proceeds in the same fashion as index building. First, each read is divided into overlapping windows of size *w*. For each read window, a *MinHash* signature is computed whose feature values are then queried against both the forward and the reverse 3 N reference index.

Each query of a feature against an index hash table yields a list of locations (sequence ID, window ID) where this feature occurs in the reference. For each read window, the candidate regions resulting from all feature queries are sorted by their sequence and window IDs which can be done with a linear time merge step since locations for each feature are stored in ascending order in the index.

We then select all locations as potential mapping candidates for a read where at least *t* many features occur in a range of windows that has at most as many windows as the read itself. The threshold *t* is important for balancing sensitivity and performance: keeping *t* high results in faster execution times since it reduces the number of alignments that have to be performed subsequently but it can also degrade mapping accuracy if set too high, because valid candidate regions might not be found (see Supplement Table 2 for experimental results analyzing this trade-off). Paired-end information (if available) is also taken into consideration by accumulating scores from neighboring windows spanning a corresponding genomic region. This results in a set of candidate regions per read which are then processed in the next step.

We exploit multi-threading on modern multi-core CPU systems by using *T* threads as follows. One thread reads sequences from file and puts them into a concurrent queue, while T/2−1 threads process the queries and generate candidate regions. Batches of these per-read candidate lists are inserted into a second concurrent queue for further processing. The remaining threads execute the steps described below by taking batches from the second queue as input. Note that read batches are evicted from memory once fully processed.

### 3.3 3N-alignment, MSA construction and inspection

Each read is aligned against all candidate regions obtained in the query step using semi-global alignment methods provided by Edlib (Šošić and Šikić 2017). Using the alignment information we then construct an MSA of a candidate region and all reads mapped to that region in the reference. This enables us to remove certain false positive mappings by inspecting the MSA, i.e. we can better distinguish true 3 N sequence conversions from sequencing errors.

MSA construction starts by loading the original (unmodified) subsequence from the reference and the original (unmodified) reads that were mapped to the considered reference region. For each reference region, sequences mapping to that region (at most 16) are put into a matrix where the candidates are shifted with respect to the reference region according to the previously obtained alignments.

These steps require loading the reference sequence(s) as well as successfully mapped read sequences into memory again, while the 3 N reference index can be evicted from memory since it is not needed any more. The reads are not loaded all at once but in batches of several GB corresponding to a contiguous range of reference sequence windows where these reads map to. Which reads map to a common reference region is determined by a list of read indices that is sorted by candidate reference region (sequence and window ID) obtained in the previous index querying step. While this might require streaming through the input read dataset a few times, it keeps the memory footprint low with only moderate runtime overhead, since computation on the current batch can be overlapped with reading the next batch of reads from disk.

In order to identify true converted nucleotide locations and distinguish them from mismatches each MSA is inspected using a combination of global and column-based scores. For each column *i* of the MSA the following statistical properties are computed: the total number of nucleotides, the percentage of As, Cs, Gs, and Ts, the most common nucleotide $M_{i}$, whether $M_{i}$ agrees with the reference, and lastly a column consensus $C_{i}$ if the relative frequency of $M_{i}$ exceeds a predefined threshold. In addition to that, statistics for each individual read in the MSA are generated which include the alignment score, the percentage of columns that deviate from the reference and the percentage of columns that deviate from the consensus $C_{i}$ if applicable. Lastly, global properties of the MSA including the total number of reads and the minimum, average and maximum alignment scores of the contained reads are calculated. All statistical properties are fed into a quality filter that discards or keeps individual read to reference region mappings. Note that this means that if a read is initially mapped to only one region that whether such a mapping is kept depends on the mappings of other reads to that same region. In case there is only one read that maps to a region, it is only filtered by a minimum alignment score threshold.

To improve the accuracy of the mapping filter, we employ a Random Forest classifier, that was pre-trained using simulated read data. Training is done by separating positive and negative case instances according to the previously described set of statistical features for each instance. During classification each individual decision tree is applied to an instance’s features, yielding a probability for each class. An instance is then classified according to the average class probabilities over the set of all decision trees. The final class label is assigned by comparing the instance’s positive class probability to a predefined threshold.

In our experiments with simulated read data we could show that the filter stage reduces the number of false positive read mappings by up to 7% and on average by 4.6% on the simulated BS-seq dataset (see Supplement Section 3 for more details on training and performance).

Finally, the resulting alignments are written to an output file in either a custom text format, which allows for faster output speed and also provides a nucleotide conversion table, or in SAM format which is widely used in many bioinformatics pipelines and also by most 3 N mappers. Like HISAT-3N we report the number of identified conversions and alignment strand (forward or reverse) using extra SAM tags. Thus, the user can obtain both the positions of the most likely conversions as well as the full alignments to the reference.

The average runtime contributions of RMapAlign3N’s stages for the *BS-seq-sim* dataset can be broken down as follows: 3 N reference index querying (12%), alignment (66%), evaluation and filtering (16%) and generation of the output that begins during the filter stage and overlaps with it and accounts for the remaining 6% of the runtime. We attribute a major part of the runtime efficiency of RMapAlign3N to the initial hash-based index querying phase. If it produces only a small number of high quality mapping candidate locations for a read, the rest of the pipeline will execute much faster since there are only few candidates to process.

In future work, we plan to make the hash-based mapping and alignment stages and the subsequent analysis stages available as two independently executable parts as well as a mode that only outputs the most likely sequence modification locations.

## Supplementary Material

vbaf164_Supplementary_Data
