# Supplementary material for: RMapAlign3N: fast mapping of 3N-Reads
Source: Bioinform Adv. 2025 Jul 9;5(1):vbaf164. doi: 10.1093/bioadv/vbaf164 (PMC12509878; doi:10.1093/bioadv/vbaf164)
Supplement: vbaf164_Supplementary_Data [file vbaf164_supplementary_data.zip › rmapalign3n_supplement.pdf]

RMapAlign3N Supplement

July 5, 2025

1 Thread Scaling Experiments

Table 1 shows the results of thread scaling experiments with RMapAlign3N, HISAT-3N, BSMAP and Bismark input read set of 100 million simulated 100-bp paired-end BS-seq reads with a 50% C-to-T conversion rate and a 0.2% per-base sequencing error rate simulated using Sherman[1].

All experiments were executed on a workstation with an AMD EPYC 7713P 64-core CPU and 512 GB of RAM.

#threads	RMapAlign3N	HISAT-3N	BSMAP	Bismark
4	121 min	417 min	636 min	3490 min
8	71 min	231 min	410 min	2316 min
16	38 min	165 min	259 min	1474 min
32	26 min	112 min	193 min	1119 min
64	15 min	67 min	129 min	899 min
128	9 min (42%)	41 min (32%)	91 min (22%)	715 min (15%)

Table 1: Runtime for different amount of CPU threads used when aligning 100 million paired-end BS-seq reads; parentheses show the parallel efficiency comparing runtimes with 128 threads vs. 4 threads

2 Mapping Sensitivity vs. Precision

Table 2 shows how alignment rate and accuracy are affected by the choice of the MinHash feature threshold t (command line parameter -hitmin) that is used to decide whether a read has sufficient similarity to a reference location to be considered as a candidate.

[1] https://github.com/FelixKrueger/Sherman

theshold t	Alignment Accuracy	Alignment Rate
2	84.10%	99.95%
3	96.32%	99.89%
4	99.70%	99.83%
6	99.79%	98.98%
8	99.82%	97.81%
10	99.82%	97.11%

Table 2: Alignment Rate and Accuracy for mapping simulated 100-bp BS-seq reads to a human reference genome when using different values for the MinHash feature threshold t used for read mapping candidate region identification.

3 Random Forest Classifier

We used the RandomForestClassifier from the scikit-learn python library and serialized the result in a custom binary format for integration into RMapAlign3N. Our random forest classifier was trained on simulated BS-seq and SLAM-seq sequencing reads with known conversions. BS-seq training data consists of datasets with 100 million BS-seq reads generated using Sherman each with different C-to-T conversion rates in the range from 10% to 90% in 10% steps and sequencing error rates of 0.1%, 0.2%, 0.3% and 0.4%. SLAM-seq training data was generated with SLAM-DUNK and consists of datasets with T-to-C conversion rates of 0.5% 1%, 2% and 4% and sequencing error rates of 0.1%, 0.2%, 0.3% and 0.4%. These datasets were only used for training.

These reads were then mapped using 3N indices, aligned and MSAs were constructed as described in the manuscript. Global MSA features used as input for the classifier (apart from positive/negative class information) include the minimum, average and maximum alignment scores of the reads, the percentages of columns that deviate from the reference, the percentage of columns that deviate from the column consensus C_i (if applicable), the total number of reads in the MSA. In addition, for each column i of the MSA the following statistical properties are used as input features: the total number of nucleotides, the percentage of As, Cs, Gs, and Ts, the most common nucleotide M_i, whether M_i agrees with the reference, and lastly a column consensus C_i if the relative frequency of M_i exceeds a predefined threshold.

Table 3 shows the percentage of false positive mappings, i.e., positions wrongly identified as conversions with and without using the random forest classifier for two read datasets (that were not used for training). For this evaluation we used the simulated BS-seq dataset with 100 million 100-bp paired-end BS-seq reads as described in the manuscript (50% C-to-T conversion rate and a 0.2% per-base sequencing error rate simulated using Sherman) as well as the simulated SLAM-seq data set with 100 million 100-bp single-end reads generated from 3' regions of transcripts with a 2% T-to-C conversion rate and a 0.2% per-base sequencing error rate simulated using SLAM-DUNK.

Mode	False Positive Conversions
BS-seq + RFC	4.6%
BS-seq w/o RFC	7.0%
SLAM-seq + RFC	5.1%
SLAM-seq w/o RFC	8.8%

Table 3: Alignment Rate and Accuracy for mapping simulated reads using the datasets described in the results section of the manuscript with and without using the random forest classifier (RFC) for MSA evaluation.

4 Commands

4.1 Database Building Commands (not all tools)

4.1.1 RMapAlign3N

```
rmapalign3n build <database_name> <reference_file1> ... <reference_fileN>
```

4.1.2 HISAT-3N

```
hisat-3n-build --3N <reference_genome> <output_tag>
```

4.1.3 Bismark

```
bismark_genome_preparation --path_to_aligner <bowtie2_directory>
    --bowtie2 <reference_genome_directory>
```

4.2 Querying Commands

4.2.1 RMapAlign3N

```
rmapalign3n query <database_name> -pairfiles <BSseq_mates1> <BSseq_mates2>
    -threads 16 -sam -out <output>
```

4.2.2 HISAT-3N

```
hisat-3n --index <index_name> -p 16 --base-change C,T --no-spliced-alignment
    -f -1 <BSseq_mates1> -2 <BSseq_mates2> -S <output>
```

```
hisat-3n --index <index_name> -p 16 --base-change T,C -f
    -U <SLAMseq_reads> -S <output>
```

4.2.3 BSMAP

```
bsmap -a <BSseq_mates1> -b <BSseq_mates2> -d <reference_genome>
    -o <output> -p 16 -n 1 -r 2 -u
```

4.2.4 Bismark

```
bismark -f --path_to_bowtie2 <bowtie2_directory> --non_directional
    --sam --bowtie2 -p 16 -genome <reference_genome_directory>
    -1 <BSseq_mates1> -2 <BSseq_mates2>
```

4.2.5 SLAM-Dunk

```
slamdunk map -r <genome_reference_file> -t 16 -o .
    -5 0 <SLAMseq_reads>
```

4.3 Read Trimming

4.3.1 Adapter Trimming:

```
trim-galore -a --length 40 -o <output_directory> <reads_file>
```

4.3.2 Poly(A)-Trimming:

```
trim-galore -a A{10} --length 40 -o <output_directory> <reads_file>
```
